# The Second Exteroceptive Suppression Period of the Temporalis Muscle Is Altered in Migraine Patients with Allodynia

**DOI:** 10.3390/neurolint17050076

**Published:** 2025-05-16

**Authors:** Eugenia Rota, Paolo Immovilli, Marco Aguggia, Maria Gabriella Saracco, Elisabetta Ghiglione, Antonella Melotti, Nicola Morelli

**Affiliations:** 1 Neurology Unit, San Giacomo Hospital, 15067 Novi Ligure, Italy; 2 Neurology Unit, Guglielmo da Saliceto Hospital, 29121 Piacenza, Italy; paolo.immovilli.md@gmail.com (P.I.); nicola.morelli.md@gmail.com (N.M.); 3 Neurology Unit, Cardinal Massaia Hospital, 14100 Asti, Italy

**Keywords:** migraine, allodynia, electrophysiology, abnormal reflex, brainstem

## Abstract

**Background/Objectives:** Studying the second exteroceptive suppression period (ES2) of the temporalis muscle may well shed some light on the brainstem neural circuits involved in migraine pathophysiology. It is known that allodynia is related to an increased sensitization of second-/third-order neurons both in the trigeminal nucleus caudalis and sensory thalamus. This pilot, observational study was carried out in the interictal period on female migraineurs with/without allodynia to assess the ES2 of the temporalis muscle compared to controls. **Methods:** Forty-nine non-consecutive female patients were enrolled, as they met the diagnostic criteria for migraine (26 episodic and 23 chronic), alongside 23 healthy controls. The inclusion criteria encompassed no ongoing pharmacological prophylactic treatment, and the exclusion criteria included any relevant comorbidities. In line with international standards, the exteroceptive suppression of the temporalis muscle activity was registered on the left side, assessing ES2 latency and duration in the interictal period. **Results:** Allodynia was observed in 24 patients (50%), and 16/24 (67%) were chronic migraineurs. No statistically significant differences in ES2 latency or its duration between the migraine patients and controls were detected. However, there was a significantly longer ES2 duration in allodynic migraineurs than in the controls (*p* = 0.04; effect size: 0.71) and in allodynic compared to non-allodynic migraineurs (*p* = 0.04; effect size: 0.63). **Conclusions:** The increased duration of ES2 observed in allodynic migraineurs might be related to the impaired activity of brainstem circuits and, in our opinion, it seems reasonable to hypothesize that this change may be a neurophysiological correlate of central sensitization in migraine allodynic patients.

## 1. Introduction

The recent literature has reported that migraine is one of the top three leading causes of disability, affecting 1.16 billion people, and that it ranks second for disability/adjusted life years among adults in Europe, with a significant physical and psychological impact and high social costs [1]. Therefore, migraine research is a must to better understand migraine pathophysiology, as it may pave the way for novel treatment regimes.

Alterations of the trigeminal nociception and of the habituation/sensitization mechanisms have been extensively detected in migraine [2]. Indeed, migraine headache attacks are underpinned by an abnormal activation and sensitization of trigeminal sensory pathways that innervate the pain-sensitive intracranial structures [3]. Due to a reduction in the nociceptive threshold, the chronic stimulation of peripheral trigeminal sensory afferents during repetitive migraine attacks leads to the sensitization of central structures of the pain pathway [4,5]. Moreover, it has been postulated that this “central sensitization” of the second-order “dural-responsive” neurons in the trigeminocervical complex and that of the third-order neurons in the thalamic sensory nuclei underpin, respectively, cephalic and extracephalic allodynia. This is a neurologic condition characterized by pain elicited by the ordinary, non-nociceptive stimulation of the skin during migraine attacks [3,6,7]. Central sensitization also occurs in patients suffering from chronic migraine in the interval phase of migraine, between attacks. This may well explain, along with allodynia, the low-grade interictal headache and other symptoms that characterize this disorder [4].

There is evidence that the trigeminal caudal nucleus also receives input from higher antinociceptive centres, which exert a descending modulation of nociceptive stimuli. These include the periaqueductal grey (PAG), the raphe magnus nucleus, and the lateral reticular nucleus [8,9,10,11] and, in turn, are controlled by higher subcortical and cortical centres that partly belong to the limbic system [11,12].

A complex dysfunction of both cortical [13] and subcortical structures has been reported in chronic migraine sufferers, including the PAG [14], which is involved in pain modulation and antinociceptive function, predisposing to the chronicization of pain.

The most common electrophysiological technique for the investigation of migraine pathophysiology is that of the elicitation of brainstem reflexes by the stimulation of the trigeminal afferents.

Electrical stimulation, delivered to the innervation territories of the second and third divisions of the trigeminus, is able to elicit both an early (first) and late (second) suppression period (ES1 and ES2, respectively) of voluntary masseter and temporalis activity. The activation of brainstem interneurons produces ES1 and ES2, inhibiting the jaw-closing muscle motoneurons: ES1 is produced via an oligosynaptic neural network and ES2 via a polysynaptic circuit [8,15].

Although there is no international consensus as to the nociceptive or non-nociceptive nature of exteroceptive suppression reflex afferents, some authors have suggested that it includes the participation of both nociceptive (Aδ and possibly C) and non-nociceptive transmitting fibres, respectively, in ES1 and ES2 [15].

Current evidence postulates that the inhibitory interneurons that mediate ES1 are to be found in the dorsal–medial pons and that they extend rostrally to the area of the main trigeminal and masticatory nuclei [16]. The location of the interneurons responsible for ES2 is less certain. However, it has been proposed that they are part of the bulbar reticular formation that lies close to the trigeminal nucleus caudalis [15,16]. This area receives afferents from both the periphery and the limbic structures, the orbito-frontal cortex, the nucleus raphe magnus, and the periaqueductal grey matter, which are relevant structures interposed in anti-nociceptive circuits [12].

**Figure 1:**
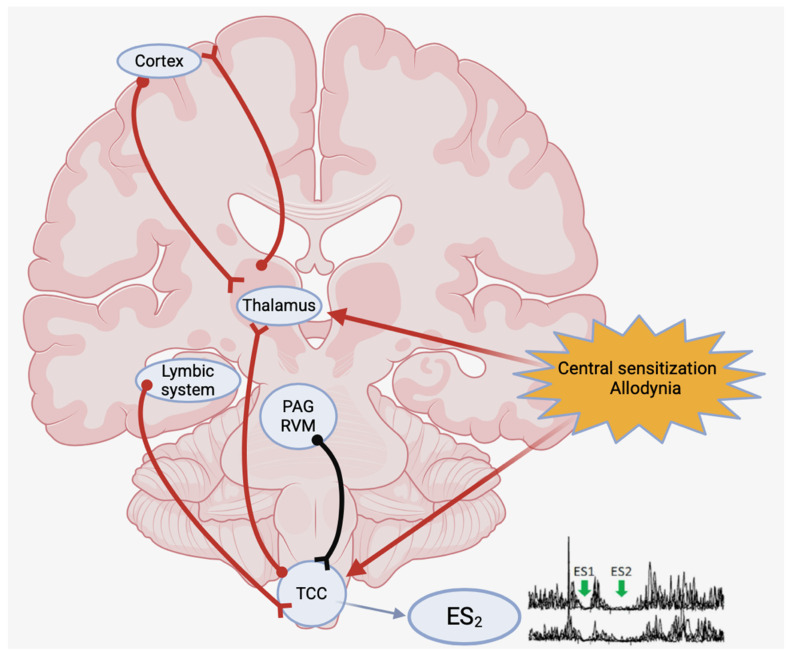
Illustrates the neural circuits which are hypothesized to be involved in the increased duration of ES2 in allodynic versus non-allodynic migraine patients and controls. The overactivity of the interneurons in the exteroceptive reflex loop may be due to the central sensitization of second-order neurons in the trigeminal nucleus caudalis and/or to concomitantly impaired inhibitory control from superior antinociceptive systems. PAG: periacqueductal grey. RVM: rostroventral medulla.

Taking into account that the interneurons responsible for ES2 receive afferents from the anti-nociceptive system and/or the trigeminal nucleus caudalis [3,4,5,12], it was decided to study the exteroceptive suppression of temporalis muscle activity, as it may well provide further information as to the functional status of the brainstem circuits involved in migraine pathophysiology, particularly those affected by the central sensitization processes that underlie allodynia. Indeed, if central sensitization can influence the excitability of trigeminal nociception, it may then be expected to modify ES2 in allodynic migraine patients (Figure 1).

This study aimed to identify any differences in the latency and/or duration of the second exteroceptive suppression period (ES2) of the temporalis muscle in a group of female migraineurs with or without allodynia and compare the data obtained to those of healthy controls. To do so, the reflex of the exteroceptive suppression of the temporalis muscle activity was investigated during the interictal period.

## 2. Materials and Methods

As aforementioned, this pilot, observational, retrospective study investigated the exteroceptive suppression reflex of temporalis muscle activity in 49 non-consecutive female migraineurs with and without allodynia and 23 healthy female volunteers. All patients gave written informed consent. The patients enrolled had been referred to our Headache Centre in a community hospital at Novi Ligure, in the Alessandria Province of Italy, for a first examination from 2020 to 2023. The exteroceptive suppression reflex of temporalis muscle activity was performed in our centre on migraineurs at the discretion of the physician.

Inclusion criteria were a diagnosis of migraine (episodic or chronic, with/without aura), according to the 3rd edition of the International Classification of Headache Disorders, volume 3 (ICHD3) [17]; age between 18 and 65; no ongoing pharmacological prophylactic treatment; and there being a time lapse of at least 72 h from the last migraine attack and/or intake of painkillers. The exclusion criteria were any internistic, neurological, or psychiatric comorbidities; the presence of a temporomandibular dysfunction or a myofascial syndrome; and/or refusal to provide written informed consent.

Exteroceptive suppression of the temporalis muscle activity was registered in line with the European Headache Federation’s standards [18], using a Natus Nicolet EDX (software Synergy 22.3). A standard bipolar stimulatory electrode was placed at the left labial commissure and 8 single stimuli were applied with a 20 mA intensity for 0.2 ms, with at least a 30 s interval between each stimulus. The EMG was recorded by two channels from the left temporalis muscle by surface electrodes (Ambu Neuroline^TM^ 715, Penang, Malaysia).

The temporalis activity was recorded by placing a disposable surface-active electrode over the anterior belly of the temporalis muscle, just before the temporal hair line, while the reference electrode was put in front of the tragus, as previously described [9].

During the stimulatory pulse application phase, the patients clenched their teeth as hard as they could.

The amplifier sensitivity was 200 μV per division with a frequency band of 20–3000 Hz. After full-wave rectification, the off-line recordings were assessed by the same investigator who was blinded as to the diagnostic category. Suppression was defined as an EMG amplitude reduction of at least 50%. According to a previously described method [19], the beginning and end of the first and second suppression periods (ES1 and ES2, respectively) of voluntary temporalis activity were defined with four cursors after the off-line recordings of the eight responses had been displayed and superimposed on the screen. Afterwards, the latency and duration of ES1 and ES2 were calculated by the same investigator.

The Allodynia Symptom Checklist (ASC-12) was used to assess the presence of cutaneous mechanical allodynia [20]. Cephalic allodynia is described as the sensitivity of the scalp and tenderness to palpation of the pericranial and cervical muscles, while extracephalic allodynia is the sensitivity of the skin or muscles in the limbs. A routine complete neurological and cranial/cervical musculoskeletal examination and a psychological assessment for allodynia and psychiatric comorbidities are given to all patients attending our Headache Centre.

The patients were asked to record the number of headache days per month in a day-by-day diary.

A descriptive statistical analysis was carried out. The clinical variables, i.e., age, monthly headache frequency, and data on temporalis ES1 and ES2 latency and duration, were evaluated by the Kolmogorov–Smirnov test and compared using Student’s *t*-test or the Mann–Whitney U test, when appropriate.

The effect size was estimated by Cohen’s d. Statistical significance was set at 0.05.

Data analyses were carried out using the Stata/MP 17.0-6 score, StataCorp LLC 4905, Lakeway Drive College Station TX 7945, USA.

## 3. Results

Forty-nine non-consecutive Italian female patients and 23 healthy female volunteers were retrospectively enrolled into the study.

Table 1 reports the main clinical and neurophysiological features, i.e., ES1, ES2, latency, and duration of the total sample, of allodynic and non-allodynic patients and controls. There was no statistically significant difference among groups (migraineurs versus healthy control, allodynic versus non-allodynic migraineurs) as to the average age (40 years for all migraine patients, 43 for allodynic, 38 for non-allodynic, and 41 for controls).

A total of 13/49 (26.5%) patients had migraine with aura; 26 (53%) had episodic migraine; and 23 (47%) had a chronic migraine pattern.

Furthermore, 24 patients (49%) had allodynia and 16/24 (67%) were chronic migraineurs.

Finally, 25 (51%) patients were non-allodynic and 4/25 (16%) were chronic.

Based on the average headache frequency in the previous three months, the average monthly migraine days were 16 in the allodynic group, 12 in the non-allodynic group, and 14 in the total migraineur sample. The average disease duration was 18 years in the allodynic group, 21 in the non-allodynic group, and 20 in the total sample of migraine patients. The average body mass index was 22.8 in the allodynic group, 22.2 in the non-allodynic group, and 22.6 in the total sample of migraine patients.

There were no statistically significant differences in the ES1 parameters or the ES2 latency between the migraine patients and controls or between allodynic and non-allodynic migraineurs. On the other hand, the ES2 duration was significantly longer in allodynic migraineurs than in the controls (*p* = 0.040; effect size: 0.71) and in allodynic migraineurs compared to non-allodynic migraineurs (*p* = 0.046; effect size: 0.63).

## 4. Discussion

This study evidenced that in the interictal period, the second exteroceptive suppression period (ES2) of the temporalis muscle lasted longer in chronic migraineurs with cutaneous mechanical allodynia, i.e., with the perception of pain in response to non-noxious skin stimulation, than it did in the controls and non-allodynic migraineurs.

We hypothesize that this may be related to impaired brainstem circuit activity, especially for the second-order neurons in the trigeminal nucleus caudalis and/or the inhibitory control from superior antinociceptive systems. Therefore, it may be reasonably supposed that this alteration in ES2 may be a neurophysiological correlate of central sensitization in migraine patients with allodynia.

Indeed, allodynia has not only been considered the clinical epiphenomenon of central sensitization in migraineurs, but it has also been linked to a higher likelihood of developing a chronic form [4], as it is more often observed in patients with frequent attacks and/or with a long-lasting disease [21].

Therefore, a better understanding of its pathophysiology and clinical correlates will hopefully improve the clinical management of migraineurs.

The mechanisms underlying cutaneous allodynia in migraine are still a question of debate. Despite this, they most likely include a sensitization phenomenon at different levels of the trigemino-vascular system and ascending projection, together with dysfunction in the various brainstem and cortical areas that modulate thalamocortical inputs [21].

Although this novel neurophysiological finding of altered ES2 in allodynic migraineurs does require confirmation in larger patient samples, it is intriguing. In fact, the literature reports evidence that the sensitization of peripheral trigeminal sensory afferents in migraine could lead to the sensitization of second- and third-order neurons in the trigeminal cervical complex and sensory thalamus. This could account for cephalic and extracephalic allodynia, respectively, during migraine attacks [3], as well as for interictal low-grade headache and allodynia [5].

It is probable that the interneurons responsible for the masseter and temporalis ES2 are located in the lateral tegmental field, near the trigeminal nucleus caudalis/cervical complex [15,16]. This area not only receives afferents from the periphery, but also from the limbic structures, the orbito-frontal cortex, the nucleus raphe magnus, and the periaqueductal grey matter, which all play a pivotal role in the endogenous control of pain. Therefore, the ES2 latency and duration may reflect, at least in part, the functionality of the brainstem circuits.

Previous studies that assessed the exteroceptive suppression of the masseter and temporalis muscle activity in primary headaches, including tension-type headache, migraine, and chronic pain, observed that ES2 was shortened or abolished in chronic tension-type headache patients [22]. Although various authors have reported contrasting results on the ES2 duration and latency in primary headaches [23], this may well depend on the differences in the methods applied and the patient samples investigated [24]. For example, the ES2 latency and duration may be affected by the migraine cycle phase, if the fact that trigeminal pain processing differs depending on whether it is studied during or outside an attack is taken into consideration [24].

In migraine, the ES2 of masseter or temporalis muscle activity has either been reported as normal [25] or as having a tendency to have a longer latency both in adults [9] and juvenile migraineurs [24]. The authors interpreted the interictal ES2 protraction observed in juvenile migraineurs as a sign of overactivity of the interneurons in the reflex loop, caused by impaired inhibitory control from superior antinociceptive systems [24].

Moreover, other neurophysiological methods, principally the blink reflex, have been employed to assess the trigeminal pathways in migraine. For example, a study on both episodic and chronic migraine patients reported an increase in R2 recovery and suggested it could be related to an impairment of the central inhibitory mechanisms during the interictal period in migraineurs [26].

Another study applied prepulse stimulation of the median nerve at the wrist [27] and observed a deficit in blink reflex sensory gating, in association with cutaneous allodynia. These observations may well support the hypothesis that cutaneous allodynia may, in part, be mediated and maintained by an abnormal sensory modulation of nociceptive afferents at the brainstem level [28,29].

The aforementioned evidence is in agreement with our results, suggesting that the increase in ES2 duration observed in our study interictally in allodynic migraineurs may be underpinned by the central sensitization of second-order neurons in the trigeminal cervical complex and/or by the impaired inhibition by the suprasegmental antinociceptive structures, including the PAG [14] (Figure 1).

Serotonin (5-HT) is able to mediate the PAG suppression of the jaw opening reflex [24]. Drugs that increase 5-HT levels reduce the ES2 duration, whilst the 5-HT receptor antagonist methysergide prolongs it [30]. 5-HT plasma levels are lower between attacks but increase during attacks [31]. Even if the underlying mechanisms involved in these changes remain to be clarified, the current data concerning the roles 5-HT play in migraine suggest that low plasma 5-HT between migraine attacks corresponds to a prolonged ES2 duration, as demonstrated both in previous studies [24] and ours.

A study on a small sample of migraineurs during erenumab treatment reported that there was a significant decrease in ES2 latency and duration in the temporalis ES2 during erenumab treatment [32].

Other research investigated the nociceptive blink reflex, and non-noxious somatosensory stimulation evoked potentials in 20 migraineurs for whom at least two preventive treatment regimens had failed, demonstrating that erenumab, along with its well-known peripheral effects, can induce central effects earlier in the brainstem and later in the cortex [33]. The nociceptive blink reflex has been suggested to be both a biomarker used to monitor central disease activity and a clinical biomarker used to predict treatment response at baseline in migraine patients [34].

Interestingly, in a recent real-world study on eighty-six patients with migraine, central sensitization, assessed by the Allodynia Symptom Checklist (ASC-12), was improved by galcanezumab [35].

These finding indicate not only that monoclonal antibodies may modulate central sensitization but also that ES2 may be a neurophysiological marker, along with the nociceptive blink reflex, of the effects these drugs might exert on the brainstem circuits involved in migraine pathophysiology [32]. Therefore, these previous findings and the results of the present study indicate the potential clinical utility of studying the second exteroceptive suppression period in relationship to the pharmacological treatment, at least in certain samples of migraine patients.

The fact that the patients were investigated in the interictal period reasonably rules out the hypothesis that peripheral sensitization affected ES2 in our study. Indeed, a neurophysiological study using a “nociception-specific” blink reflex demonstrated temporary sensitization of central trigeminal neurons during acute migraine attacks, which revealed a shortening of R2 onset latencies [36].

However, we are aware that this study does have some limitations, such as the small number of cases and our having studied only female migraineurs; therefore, the generalizability of the findings might be limited due to the restricted sample size. Moreover, there was a wide age range for the patients in the study.

The neurophysiological investigation of the masseteric and temporalis exteroceptive suppression reflex is known to pose numerous technical challenges. Despite this, we did our best to minimize the technical factors responsible for the variability reported in the literature regarding normal values for ES2 duration, which has sometimes been attributable to subtle differences in methods [24].

Moreover, the interpretation of some ES2 suppression periods may be uncertain, as such a response is mediated by a multisynaptic circuit and therefore may be influenced by both the patient’s cooperation and mandibular function [16].

Furthermore, as the exteroceptive suppression period of the temporalis muscle was registered only on the left-hand side, it cannot be ruled out that if the side where the prevalent pain was located had also been investigated, the results may have differed.

Lastly, although ES2 may vary due to hormonal factors, the phase of the menstrual cycle was not taken into account when ES2 registration was carried out [24]. Indeed, over time, individual physiologic fluctuations and the use of various methods have been recognized as major limitations of electrophysiological research in migraine [37].

However, despite these drawbacks, in our study, there was a statistically significant difference in the temporalis ES2 duration in allodynic migraineurs compared to non-allodynic ones and the controls. This finding may be related to the central sensitization of brainstem trigeminal nuclei and/or impaired suprasegmental inhibitory neural circuits involved in migraine pathophysiology (Figure 1).

Even though there is no doubt that neurophysiological techniques are very useful in the investigation of the pathophysiological basis of migraine, particularly migraine with aura [38], further studies are warranted to confirm this intriguing hypothesis.

## 5. Conclusions

The modification of the temporalis ES2 duration in allodynic migraine patients versus non-allodynic patients and controls observed in this study seems to indicate that ES2 does have a role to play as a neurophysiological marker of central sensitization associated with allodynia during the interval period in chronic migraineurs. Indeed, such an interictal ES2 protraction suggests overactivity of the interneurons in the reflex loop due to sensitized second-order neurons in the trigeminal nucleus caudalis and/or impaired inhibitory control from superior antinociceptive systems (Figure 1).

Hopefully, further studies on larger patient samples will confirm these preliminary findings in the context of such an intriguing hypothesis and the potential clinical applications of the second exteroceptive suppression period of the temporalis muscle in migraine management. Indeed, the data herein reported indicate that the study of ES2 would be a useful tool to investigate the functionality of brainstem circuits in migraine patients.

## Figures and Tables

**Table 1:** The main clinical and neurophysiological features (ES1, ES2, latency, and duration; average, msec) of migraineurs, including allodynic and non-allodynic migraineurs, and healthy controls; comparison (using Student’s *t*-test or the Mann–Whitney U test, when appropriate) between migraineurs and controls, allodynic and non-allodynic migraineurs, and allodynic migraineurs and controls.

Allodynic vs. Controls	Allodynic vs. Non-allodynic	Migraineurs vs. Controls	Controls (N°: 23)	Non-Allodynic (N°: 25)	Allodynic (N°: 24)	Migraineurs (Total N°: 49)	
p: 0.63	p: 0.51	p: 0.44	41 (20–61)	43 (18–62)	38 (22–59)	40 (18–62)	Age (average, years) (min–max value)
-	p: 0.63	-	-	12 (2–26)	16 (3–30)	14 (2–30)	Headache monthly frequency (min–max value)
p: 0.31	p: 0.75	p: 0.62	13.9 ± 1.3 (11–16.8)	12.6 ± 1.9 (9.6–15.2)	13.1 ± 1.3 (11–15.2)	12.8 ± 1.5 (9.6–16.2)	ES1 latency (average, msec) ± SD (min–max value)
p: 0.21	p: 0.94	p: 0.12	14.1 ± 3.1 (7.8–17.6)	13.8 ± 3.0 (10.4–18.5)	14.9 ± 4.7 (10–17.4)	14.4 ± 3.4 (10–18.5)	ES1 duration (average, msec) ± SD (min–max value)
p:0.44	p: 0.69	p: 0.61	56.8 ± 7.2 (49.9–66)	60.1 ± 9.7 (55.1–76.9)	62.1 ± 11.1 (42.2–98.8)	61.5 ± 8.5 (42.2–98.8)	ES2 latency (average, msec) ± SD (min–max value)
**p: 0.040**	**p: 0.046**	p: 0.78	25.6 ± 7.4 (21.2–32.8)	26.5 ± 6.4 (21.4–28.2)	31.8 ± 10.0 (24–43.8)	28.2 ± 7.7 (21.4–43.8)	ES2 duration (average, msec) ± SD (min–max value)

## Data Availability

Data are not available for internal regulations in our Institution.
